# Optimisation of three-dimensional lower jaw resection margin planning using a novel Black Bone magnetic resonance imaging protocol

**DOI:** 10.1371/journal.pone.0196059

**Published:** 2018-04-20

**Authors:** Astrid M. Hoving, Joep Kraeima, Rutger H. Schepers, Hildebrand Dijkstra, Jan Hendrik Potze, Bart Dorgelo, Max J. H. Witjes

**Affiliations:** 1 Department of Oral and Maxillofacial Surgery, University Medical Centre Groningen, Groningen, The Netherlands; 2 Department of Radiology, University Medical Centre Groningen, Groningen, The Netherlands; Public Library of Science, UNITED KINGDOM

## Abstract

**Background:**

MRI is the optimal method for sensitive detection of tumour tissue and pre-operative staging in oral cancer. When jawbone resections are necessary, the current standard of care for oral tumour surgery in our hospital is 3D virtual planning from CT data. 3D printed jawbone cutting guides are designed from the CT data. The tumour margins are difficult to visualise on CT, whereas they are clearly visible on MRI scans. The aim of this study was to change the conventional CT-based workflow by developing a method for 3D MRI-based lower jaw models. The MRI-based visualisation of the tumour aids in planning bone resection margins.

**Materials and findings:**

A workflow for MRI-based 3D surgical planning with bone cutting guides was developed using a four-step approach. Key MRI parameters were defined (phase 1), followed by an application of selected Black Bone MRI sequences on healthy volunteers (phase 2). Three Black Bone MRI sequences were chosen for phase 3: standard, fat saturated, and an out of phase sequence. These protocols were validated by applying them on patients (n = 10) and comparison to corresponding CT data. The mean deviation values between the MRI- and the CT-based models were 0.63, 0.59 and 0.80 mm for the three evaluated Black Bone MRI sequences. Phase 4 entailed examination of the clinical value during surgery, using excellently fitting printed bone cutting guides designed from MRI-based lower jaw models, in two patients with oral cancer. The mean deviation of the resection planes was 2.3 mm, 3.8 mm for the fibula segments, and the mean axis deviation was the fibula segments of 1.9°.

**Conclusions:**

This study offers a method for 3D virtual resection planning and surgery using cutting guides based solely on MRI imaging. Therefore, no additional CT data are required for 3D virtual planning in oral cancer surgery.

## Introduction

Patients suffering from a malignant or benign oral tumour involving the lower jaw, are often treated with a partial lower jaw resection followed by a reconstruction of the bone defect using autologous tissue transfer. Bone is often harvested from the fibula (lower leg) and transplanted to the lower jaw defect. This so called fibula free flap reconstruction is a standard procedure in reconstruction of jaw bone defects. In the UMCG, the current standard of care in head and neck oncology is three-dimensional (3D) virtual planning of resection and reconstruction followed by surgery with the use of 3D printed bone cutting guides [1,2]. The factor of success in this current workflow is the integration of tumour margins derived from MRI data into the surgery plan through 3D models derived from computed tomography (CT) data. Magnetic resonance imaging (MRI) data delineate tumour margins better than CT [3]. Both CT and MRI are used for reliable diagnosis and surgical planning [1]. The workflow necessitates MRI and CT data fusion, since actual fusion of CT and MRI data is more accurate than visual (on screen) comparison of the two modalities. Eventually, this approach results in the inclusion of tumour margins in the pre-surgical plan. The tumour is directly visualised in the CT-based 3D model, which provides the possibility of placing the bone cutting planes and the position and design of the cutting guides based on the tumour margin.

The multimodality data fusion has an accuracy error of 1–2 mm [4–11]. This introduces additional inaccuracies in resection margin planning, which could lead to incomplete removal of tumorous tissue. Furthermore, lower jaw resections that are executed with 3D printed surgical guides also show an average deviation of 2 mm from the original plan [2]. A planning workflow based on a single modality would make the CT-MRI data fusion step superfluous thereby eliminating the corresponding accuracy error from the surgical plan. MRI is the most promising for a single-image-modality planning workflow, since both tumour and bone information can be retrieved from MRI data. Several studies report accurate 3D bone models from other anatomic areas derived from MRI data [12–24]. To come to a single modality MRI planning, it is necessary to derive 3D bone models from MRI images that are as accurate as bone models from CT.

The aim of this study is to offer an alternative for the current CT-MRI-based workflow for lower jaw resection and reconstructive surgery planning in oral cancer surgery. A method is developed to obtain 3D MRI-based lower jaw 3D models for tumour resection via a single modality planning workflow.

## Materials and methods

The study design was approved by the local medical ethics committee (Medisch Ethische Toetsingscommissie van het Universitair Medisch Centrum Groningen (METc UMCG)) under number M16.198347.

**Fig 1:**
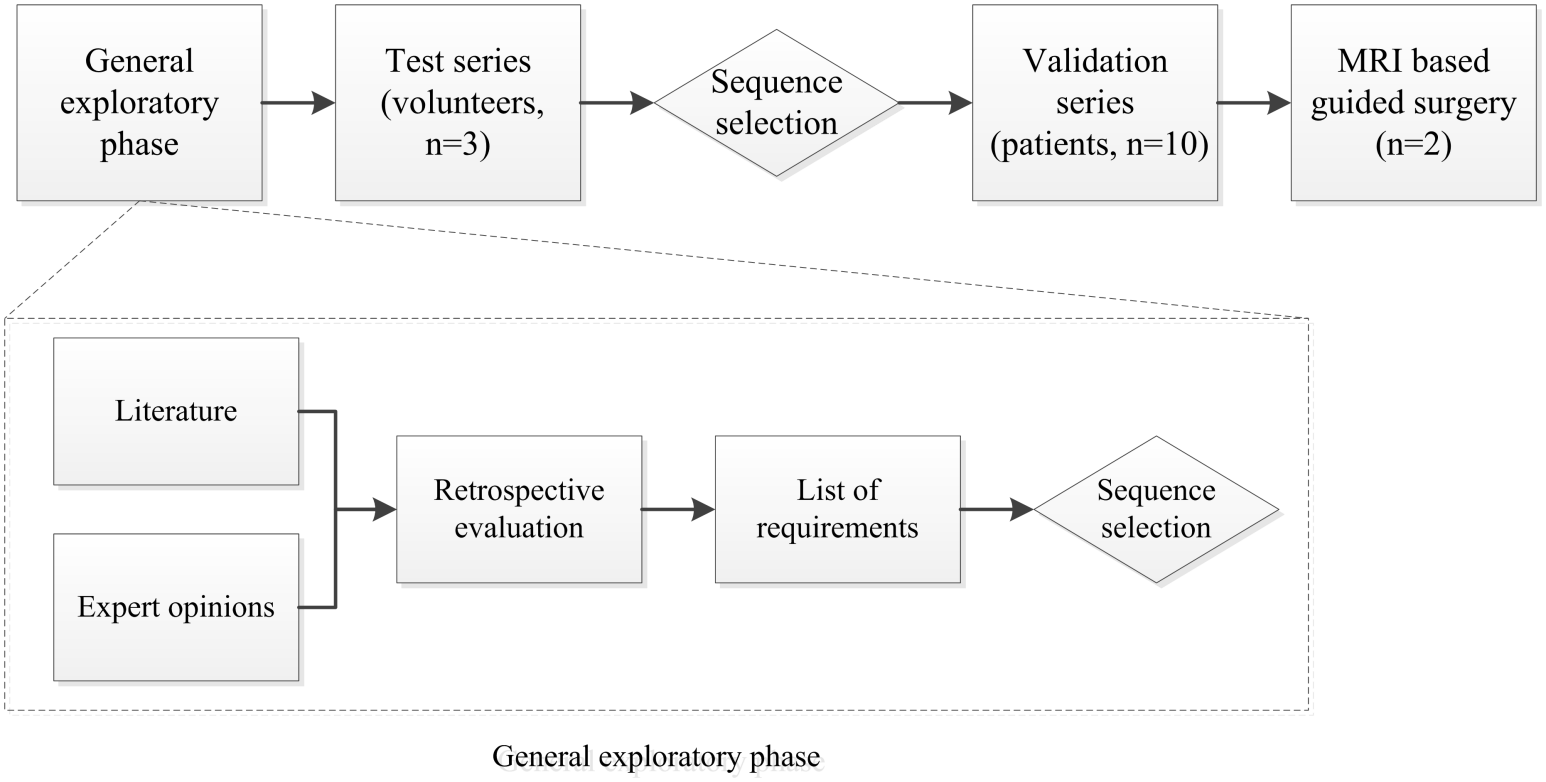
Schematic outline of the workflow of this study. The four phases are: general exploratory phase, test series, validation series, and MRI-based surgery. The rhombic boxes show decision making moments in the workflow.

A four phase approach was established to obtain 3D MRI-based lower jaw models: a) general exploratory phase to define essential MRI related parameters for bone segmentation, b) test series of the most relevant MRI settings, c) validation series of the selected MRI parameters, and d) MRI-based virtual planning for tumour-surgery of an oral squamous cell carcinoma (Fig 1).

### General exploratory phase

A literature search was performed to find the key MRI parameters for bone segmentation. Web of Science was accessed to search the following terms: “bone segmentation MRI”, “3D bone model MRI”, and “mandible bone segmentation MRI”. The key MRI parameters were selected after an interdisciplinary consultation between a technical physician, a radiologist and an MRI technician (A.H., B.D. & J.H.P). The key MRI parameters were included in the retrospective analysis of the MRI case data.

MRI data of the head and neck regions, scanned with the selected MRI parameters, were collected retrospectively (n = 13). The lower jaw was segmented from the MRI data using Mimics Medical 18.0 (Materialise, Leuven, Belgium). The segmentation quality of eight different MRI sequences were compared: T1 3D VIBE, 3D MPRAGE, T1 TSE, T2 3D FLAIR + FATSAT, T1 starVIBE + Gadolinium contrast medium, T2 blade, T1 3D Dixon VIBE, and Black Bone VIBE (with and without Gadolinium contrast medium (Dotarem^®^, Guerbet). A 3 Tesla MRI-scanner (MAGNETOM Prisma, Siemens, Erlangen, Germany) was used with a 64-channel head and neck coil. The quality of the 3D lower jaw models generated from the MRI data was evaluated by focusing on the following aspects: contrast between lower jaw and surrounding tissue, (3D) scanning protocol, time of segmentation, and quality of 3D lower jaw models (compared to CT-based 3D lower jaw models when available). The literature study and the quality of the obtained 3D models of the lower jaw, defined the requirements for MRI sequences, scan resolution and slice thickness as well as suitable segmentation methods for 3D planning. One was selected from the eight MRI sequences for further optimisation and validation.

### Test series

To optimise the selected MRI sequence, test series were performed on three healthy volunteers (2 male, 1 female, mean age: 29 years). The Black Bone sequence, as described by *Eley et al*. [25], acted as a starting point. In the optimisation process, the flip angle was modified, followed by the addition of fat saturation (quick FATSAT), scanning with/without interpolation, addition of GeneRalized Autocalibrating Partial Parallel Acquisition (GRAPPA), and out of phase scanning. The flip angles varied between 2° and 7°. Not all sequences were performed on all volunteers, because interim analysis gave us insights to test other scan parameters. A short explanation of the MRI terms is given in Table 1.

**Table 1:** Short explanation of four important MRI parameters.

Term	Description	Result on image
**Flip angle (α)**	The angle at which the longitudinal magnetisation flips in the xy-plane at excitation.	A very small flip angle (α<10°) will result in a more homogeneous image with less contrast between soft tissues.
**Quick FATSAT**	Immediately after the protons bound to fat are excited, a spoiler gradient destroys the fat signal.	The water bound protons are visualised bright, the fat bound protons are black.
**GRAPPA**	Faster acquisition is possible by using a limited number of phase encoding steps.	Impaired image quality due to more noise (worse signal-to-noise-ratio).
**Out of phase**	Opposed phase imaging makes use of the difference in resonance frequencies of water and fat.	Opposed phase images show sharply black lines around organs with a fat-water interface.

All the series were performed with a 3T MRI-scanner (MAGNETOM Prisma, Siemens, Erlangen, Germany) using a 64-channel head and neck coil. The field of view (FOV) was set onto the entire affected lower jaw area, but not more. Additional fixation of the head was accomplished with foam pillows or towels. The volunteers were positioned supine and head first, conforming to conventional protocol. The standard sequence was a Black Bone VIBE sequence with interpolation. Voxel size was 0.7 mm for all series. S1 Table shows the sequence parameters utilised in this study’s test phase.

The Black Bone MRI scans were evaluated by segmenting the lower jaw and the 3D model was assessed using Mimics Medical 18.0. The following smoothing settings were applied: smoothing factor 0.8, iterations 5, and compensate for shrinking.

The quality of the 3D models was utilised to select the optimal Black Bone sequence. Each 3D model was scored by one observer (A.H.) using a quality scale-based scoring system. Anatomical ROIs for 3D cutting guide designs were defined based on a retrospective evaluation of seventeen lower jaw cutting guide designs that had been used previously in oral cancer surgery where a part of the lower jaw was removed. This led to the definition of three ROIs for quality evaluation. For each ROI, a scoring (++, +, -, —) was assigned to the number of virtual holes (mental region, left and right lower jaw angles) and the number of parts edited (manual editing). The three best scoring sequences were chosen for use in the validation series.

### Validation series

Three selected MRI Black Bone sequences were validated using patient data. The goal of the validation phase was to select the most promising Black Bone sequence settings for 3D modelling of the lower jaw via surface comparison of CT- and MRI-based models.

Ten patients (mean age: 67.8 (min-max 50–81), 5 male) with oral cancer and undergoing MRI and CT imaging of the tumour as part of the diagnostic work up were selected prospectively. The three Black Bone sequences were added to the conventional MRI protocol, which increased scanning time by nine minutes. All series were performed with a 3T MRI-scanner (MAGNETOM Prisma, Siemens, Erlangen, Germany) using a 64-channel head and neck coil. The patients were positioned supine and head first. Table 2 shows the three Black Bone sequences with their parameters. The flip angle was 2° and the voxel size was 0.7 mm in all sequences.

**Table 2:** Sequences and characteristics of the validation series.

No.	Series description	BW	TR	TE	TA
**1**	Black Bone with quick FATSAT + GRAPPA	210	6.2	2.53	2:38
**2**	Black Bone out of phase + GRAPPA	530	3.78	1.54	1:34
**3**	Black Bone standard	210	6.2	2.53	4:42

The sequences are all variations of the Black Bone sequence. BW = pixel bandwidth (Hz/pixel), TR = repetition time (ms), TE = echo time (ms), TA = acquisition time (min)

The test series method was utilised for the segmentation of the lower jaw. CT data was segmented using a predefined bone threshold (HU value range: 1500–4095) in Mimics Medical 18.0. 3D models were calculated with the same settings as used for the MRI calculations. The CT- and MRI-based 3D models were exported as binary STL files.

The quality of the 3D MRI-based models was scored according to the method used in the test series. Moreover, a quantitative surface comparison of the 3D MRI- and CT-based models was done. First, an iterative closest point (ICP) algorithm in Geomagic Studio 2012.0.0 (Geomagic GmbH) was applied to gain a surface match. Then, two planes were defined in both the aligned MRI- and CT-based models which were going to be used to remove parts of the lower jaw, using 3-Matic Medical 10.0 (Materialise, Leuven, Belgium), whilst maintaining the anatomical ROIs. The analysis was completed by a part to part comparison. Mean deviation and distance maps were calculated to show the difference between the CT and MRI-based models.

### MRI-based 3D planned surgery

Guided tumour resection with 3D printed cutting guides designed on MRI-based virtual lower jaw models, was performed on two patient cases from the validation series. The two patients (age: 67, 81, both female) suffered from oral cancer and the individual surgical plans included removal of a part of the lower jaw.

The two cases were both diagnosed with oral cavity T4 squamous cell carcinoma, requiring resection of the lower jaw followed by (segmented) fibula reconstruction of the defect as described by the Dutch Guidelines for oral cancer treatment. The diagnostic and tumour surgery workups were not altered from the conventional workflow. The protocol includes both T1 and T2 images and the use of Gadolinium contrast agent. Mirada software (Mirada Medical, Oxford Centre for Innovation, United Kingdom) is utilized to manually delineate gross tumour volume (GTV) on the MRI data. The tumour delineation is performed by the involved technical physician and validated by the radiologist and maxillofacial surgeon. This protocol was followed by the preoperative 3D virtual planning procedure [1]. All three sequences were evaluated for both patients, the best sequence per case was chosen to perform the surgical planning on. MRI data were used for the 3D bone modelling and resection planning using ProPlan CMF 1.3 (Materialise, Leuven, Belgium).

To evaluate the accuracy of the MRI-based models and the suitability for an MRI-based patient specific reconstruction plate (PSP), PSP test designs were made using 3-Matic Medical 10.0. The MRI-based plates were evaluated in 3-Matic Medical 10.0 by fitting the plates virtually onto the CT-based lower jaw model. Additionally, the MRI and CT-based 3D models and the test plates were 3D printed and evaluated by experienced maxillofacial surgeons. The PSP (one case) and accessory resection guides (both cases) to be used in surgery were designed based on MRI data and produced by KLS Martin. At the UMCG, not every patient is treated with a PSP, it depends on the location and type of reconstructive surgery. A one segment fibula in an edentulous patient generally does not require a PSP. A 3D PSP print with locking screw holes was made for the first case. The surgical outcome was evaluated during surgery by fitting the guides and the PSP to the jaw. To evaluate the surgical accuracy, the 3D model of the post-operative cone beam CT (CBCT) scan and the MRI-based surgical plan were aligned virtually using Geomagic studio 12.0.0. Moreover, the orientation of the fibula segments (planned vs. post-operative) were assessed. Midpoints and direction vectors of the segments and planes were compared using the method described by *Schepers et al*. *2015* [2].

A written consent form was obtained with regard to any patient data used in this manuscript.

## Results

### General exploratory phase

The literature search yielded 24 papers describing MRI bone segmentation (S2 Table). These studies all used 1.5T or 3T MRI devices (Siemens, Philips, or GE). Commonly used sequences were 3D T2-weighted gradient echo, T1-weighted FLASH, VIBE, spoiled gradient echo sequences and MPRAGE. Some studies used additional water-fat separation, water selective excitation or fat suppression techniques. Pixel sizes ranged from 0.1×0.1–1.3×1.3 mm^2^ with slice thicknesses in the range of 0.5–5.0 mm. Isotropic voxel sizes were reported in a few studies.

A list of requirements, based on the literature search and segmentation of several MRI sequences, is described in S3 Table. The 3D Black Bone VIBE sequence was selected as a starting point for further optimisation. A detailed description of the segmentation examples and the list of requirements are given in the supporting files (S4 Table, S1–S6 Figs).

### Test series

Table 3 shows the quality assessment of the lower jaw bone segmentations from the three volunteers. Inter-subject differences were found between the 3D models of the same sequences.

**Table 3:** Segmentation quality scoring of each 3D lower jaw model derived from the test series.

No.	Series description	Volun-teer	Mental region	Left lower jaw angle	Right lower jaw angle	Manual editing
**1**	FA 2	1	--	+	+	+
		2	+	+	+	+
		3	--	-	++	+
**2**	FA 3	1	--	+	++	+
		2	+	+	+	+
**3**	FA 5 out of phase	1	+	+	+	+
		2	+	+	+	++
		3	+	--	**-**	+
**4**	FA 5 with quick FATSAT	1	+	+	+	-
		2	+	+	+	+
		3	--	+	+	-
**5**	FA 5	1	--	+	++	-
		2	-	+	+	+
		3	-	+	++	+
**6**	FA 5 without interpolation	1	--	+	+	+
		2	-	+	+	++
**7**	FA 7	1	-	+	++	+
		2	--	+	+	++
**8**	FA 5 without interpolation + GRAPPA	2	+	+	+	++
**9**	FA 2 out of phase + GRAPPA	3	+	--	-	+
**10**	FA 2 out of phase + GRAPPA3	3	+	--	-	+
**11**	FA 2 out of phase	3	+	--	-	++
**12**	FA 2 with quick FATSAT	3	--	+	+	-
**13**	FA 2 with quick FATSAT + GRAPPA	3	--	+	+	-
**14**	FA 2 with quick FATSAT +GRAPPA3	3	--	+	+	-
**15**	FA 2 + GRAPPA 3	3	-	+	++	+
**16**	FA 5 out of phase + GRAPPA	3	+	--	-	+
**17**	FA 5 with quick FATSAT + GRAPPA	3	--	+	+	-

FA = flip angle. Mental region and lower jaw angles/manual editing:—one large hole or multiple large holes/more than 15 parts edited;—multiple holes/10-15 parts edited; + view holes/5-10 parts edited; ++ no holes/less than 5 parts edited.

**Fig 2:**
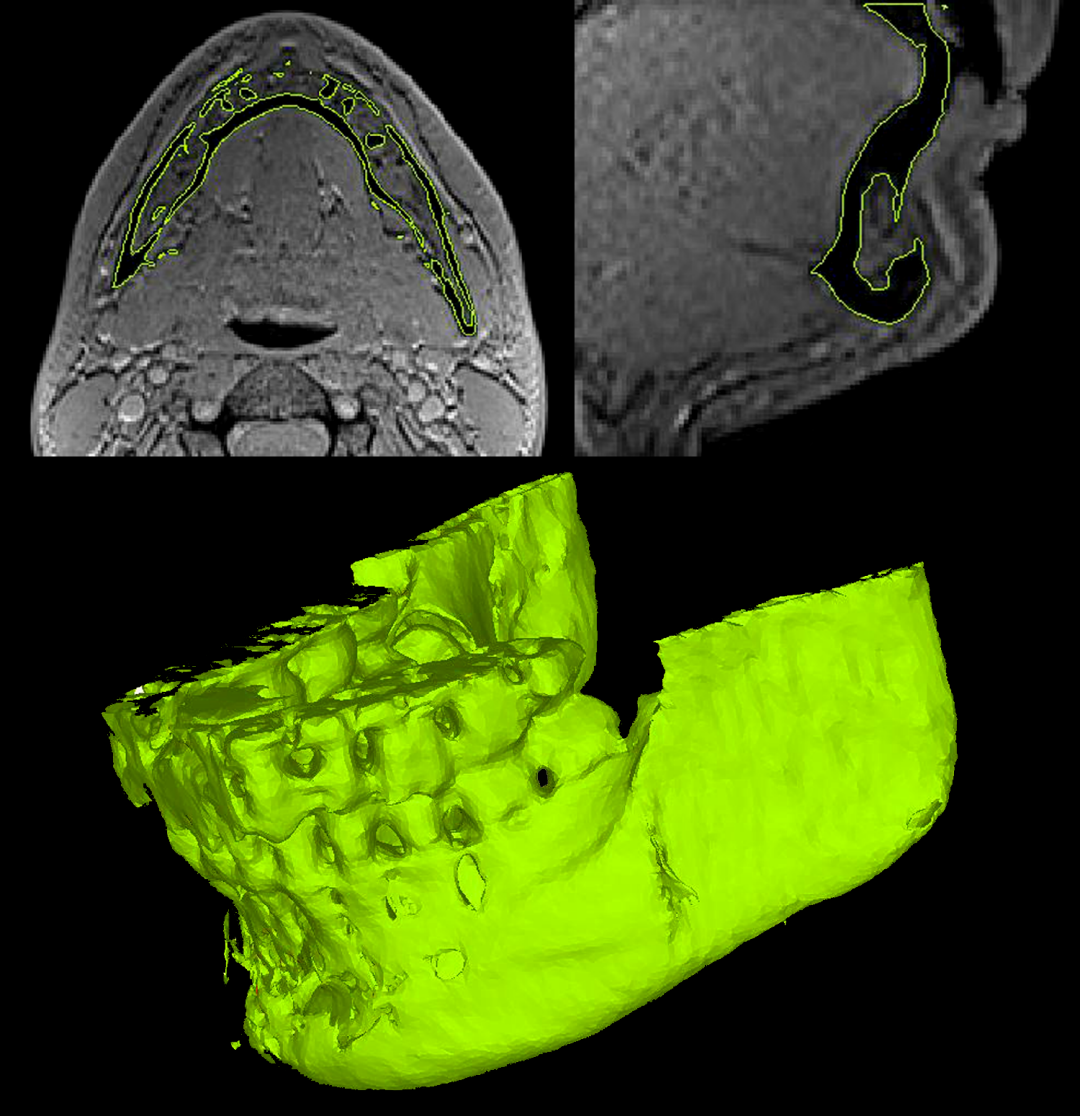
Impaired segmentation quality of the mental region. The axial and sagittal slices and the 3D model derived from the standard Black Bone MRI sequence (with a flip angle of 2°) show the impaired segmentation quality of the mental region. The interruption in the black boundary (cortical bone) creating the virtual holes in the model is visible in the slices.

**Fig 3:**
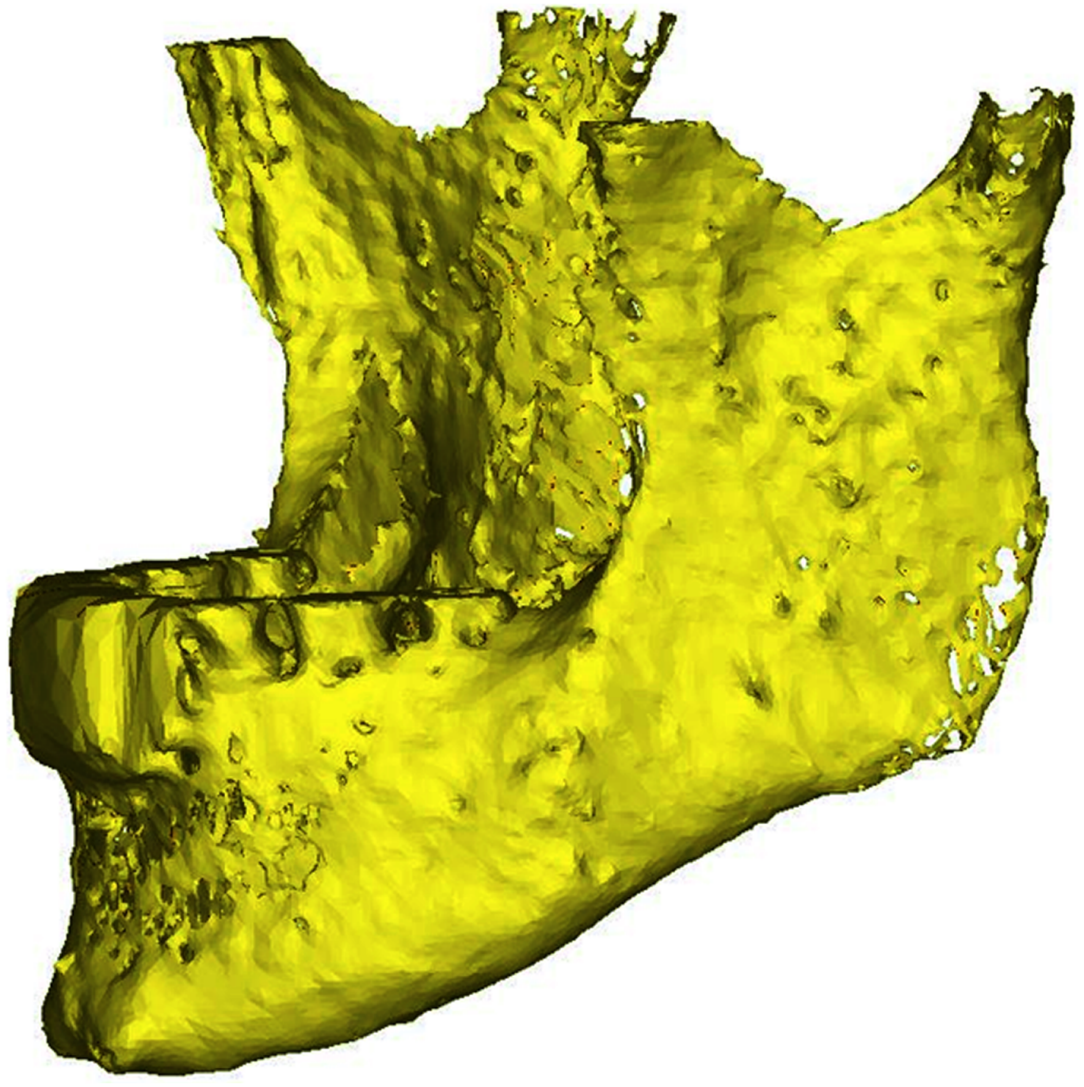
Impaired lower jaw angle segmentation quality. Impaired segmentation quality (virtual holes) is visible in the left lower jaw angle in the 3D model derived from the out of phase Black Bone sequence with a flip angle of 5° and GRAPPA settings.

Despite the reported intra-subject inconsistencies, the quality of the segmentations was generally similar. The Black Bone sequences without the use of quick FATSAT or out of phase imaging showed poor segmentation quality in the mental region (Fig 2). The images showed interruptions of the black boundary in that area. The sequences with quick FATSAT settings generally needed more manual editing to remove additional attachments, whereas the out of phase sequences needed the least manual editing. The trade-off here is that the segmentation quality of the lower jaw angles is impaired (Fig 3). The influence of GRAPPA and GRAPPA3 was not visible in the segmentation quality.

Quick FATSAT, out of phase, and standard Black Bone imaging were selected for the validation series in a larger (patient) group.

### Validation series

**Fig 4:**
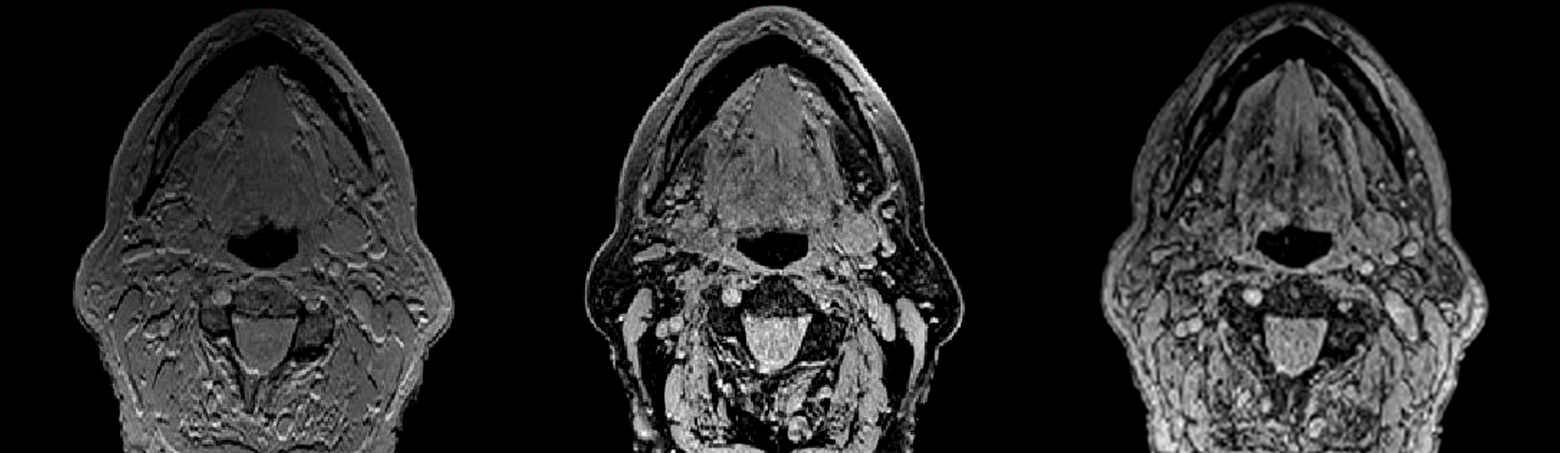
Axial views of the three different Black Bone sequences. From left to right: standard Black Bone MRI sequence, Black Bone with quick FATSAT + GRAPPA, and Black Bone out of phase + quick FATSAT. More noise is visible in the middle and right images and these images also show more soft tissue contrast.

Most scans required manual editing resulting in a segmentation process of about 30–60 minutes. In contrast to the results of the test series, the standard scan (Black Bone sequence without quick FATSAT or out of phase scanning) of most of the cases in the validation series required the least manual editing (Table 4). The series performed with GRAPPA showed an increased amount of noise compared to the images obtained without GRAPPA. These series also showed increased soft tissue contrast (Fig 4).

**Table 4:** Segmentation quality scoring of each 3D lower jaw model derived from the validation series.

No.	Series	Mental region	Left lower jaw angle	Right lower jaw angle	Manual editing
**1**	A	+	-	-	--
	B	+	+	+	--
	C	+	++	++	-
**2**	A	++	+	++	-
	B	++	-	+	-
	C	+	+	++	+
**3**	A	+	+	+	--
	B	+	-	+	-
	C	--	-	-	--
**4**	A	-	-	+	--
	B	++	+	+	--
	C	--	-	+	--
**5**	A	+	+	+	--
	B	+	+	+	--
	C	++	++	++	+
**6**	A	+	+	+	--
	B	+	-	-	--
	C	+	+	+	+
**7**	A	++	+	++	--
	B	++	+	+	--
	C	-	+	++	+
**8**	A	+	-	-	-
	B	+	-	-	--
	C	++	--	-	-
**9**	A	++	++	++	-
	B	++	+	++	-
	C	++	+	++	+
**10**	A	+	+	+	--
	B	+	+	+	--
	C	+	+	+	+

A = FA 2 FATSAT with GRAPPA, B = FA 2 out of phase with GRAPPA, C = FA 2. Mental region and lower jaw angles/manual editing:—one large hole or multiple large holes/more than 15 parts edited;—multiple holes/10-15 parts edited; + view holes/5-10 parts edited; ++ no holes/less than 5 parts edited.

The average error of the alignment measurements of CT- and MRI-based models indicates the average deviation of all points of comparison. This indicates the accuracy of alignment. The models are aligned within an average accuracy of 0.7 mm.

**Fig 5:**
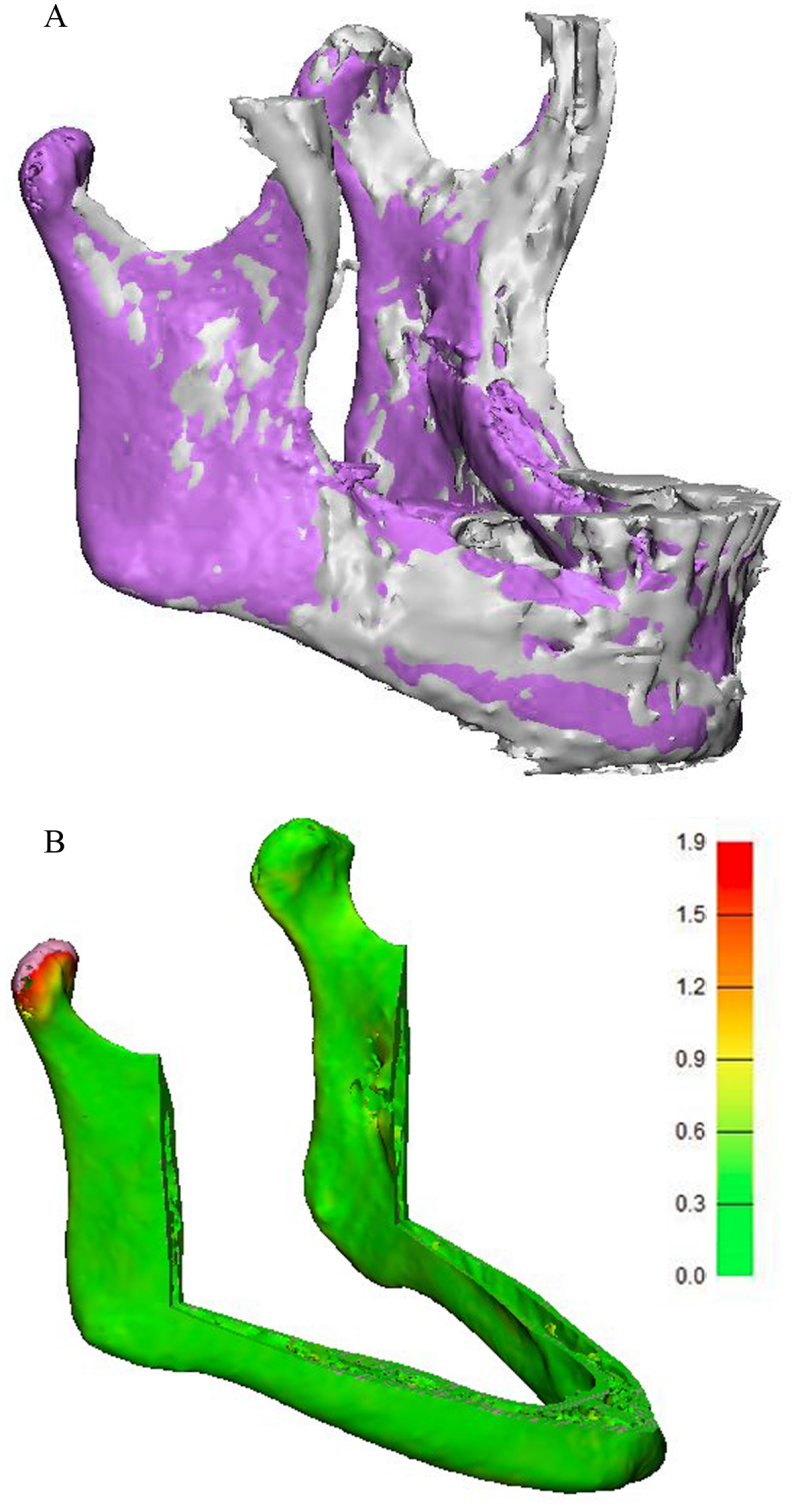
Aligned CT- and MRI-based models. (A) Aligned CT- (purple) and MRI-based (grey) models. (B) Distance map of the reduced models. The CT-based model is analysed and compared to the MRI-based model.

The mean deviation values between the reduced MRI-based models and the CT-based models are between 0.6 and 0.8 mm for all three sequences (Table 5). Fig 5 shows examples of aligned CT- and MRI-based models and the reduced models.

**Table 5:** Mean values and standard deviation of the deviation analysis measurements between CT- and MRI-based models.

Sequence	Mean deviation in mm (stdev)
**FA 2 with quick FATSAT + GRAPPA**	0.63 (0.58)
**FA 2 out of phase + GRAPPA**	0.59 (0.55)
**FA 2**	0.80 (0.88)

This analysis is performed after reducing the models by two cutting planes, whilst maintaining the ROI for comparison purposes.

### MRI-based 3D planned surgery

**Fig 6:**
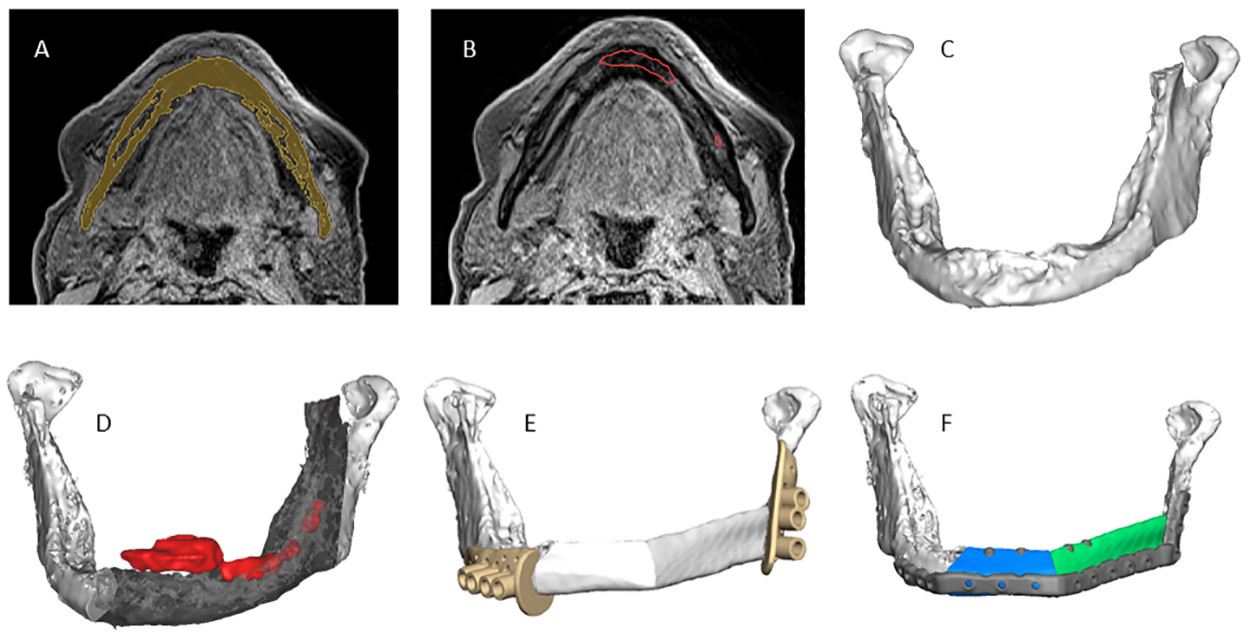
MRI-based 3D virtual planning workflow of the first case. (A) Segmentation of the mandible model (yellow) from Black Bone MRI. (B) Delineated tumour contour (red) projected onto Black Bone MRI. (C) MRI-based 3D model. (D) MRI-based 3D model including tumour outline (red) and bone to be resected (semi-transparent grey). (E) Design of resection guides and reconstruction with fibula. (F) Final reconstructive planning, including patient specific osteosynthesis plate.

The most adequate segmented sequence was selected for a patient with a T4 oral tumour, a Black Bone with quick FATSAT + GRAPPA and a flip angle of 2°, for patient specific reconstruction plate (PSP) design and surgical margin planning. The workflow for this case is shown in Fig 6. The second MRI-based surgery used the Black Bone sequence with a flip angle of 2° (S7 Fig).

The virtual evaluation of the test plate showed the MRI-based plate fitted properly (S8 Fig). The test plate deviated slightly from the model in the mental region, as well as in the region where the masseter muscle overlaps the lower jaw (both right and left side), and it deviated minimally on top of both the left and right ramus in terms of an anterior opening between plate and bone surface. The planning of the surgery was decisively based on the virtual fitting of the test plate on the MRI-based models together with the expert opinion of two surgeons.

**Fig 7:**
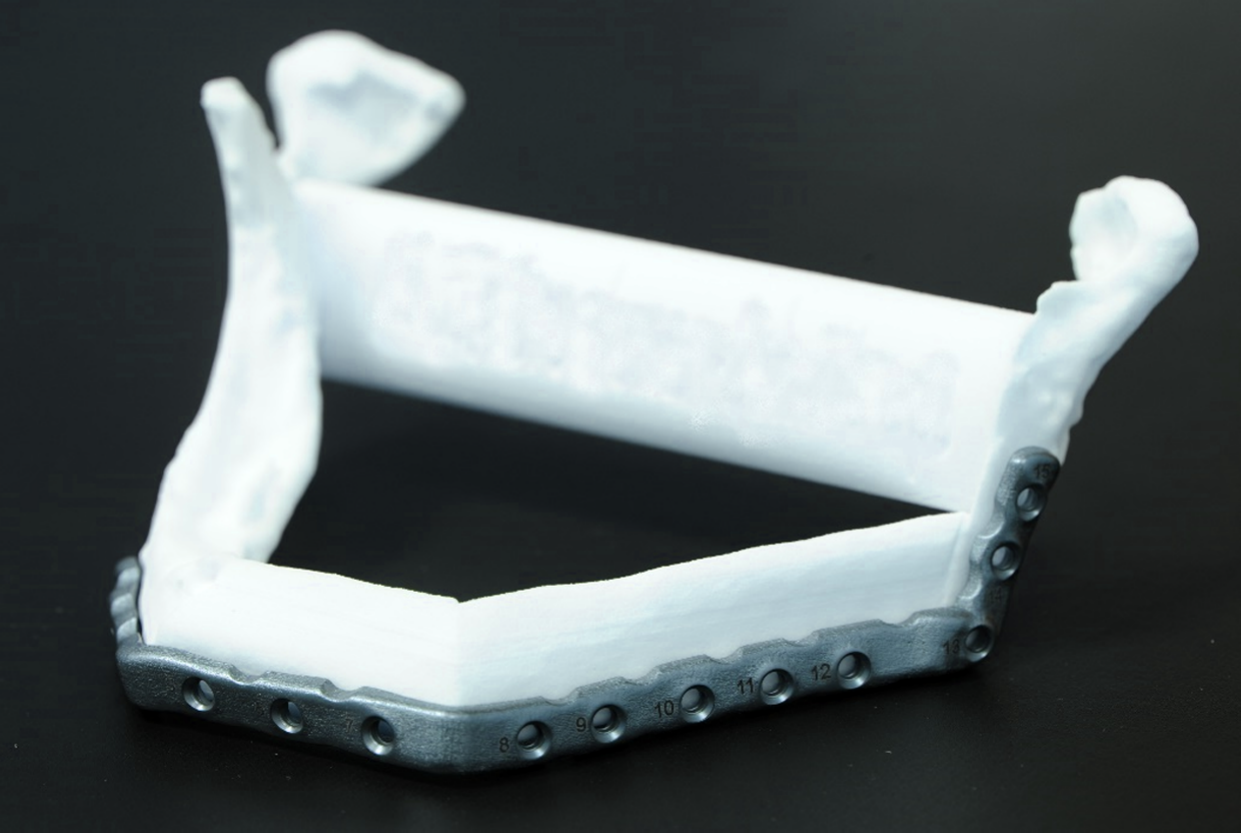
3D printed patient specific reconstruction plate (PSP). The 3D printed plate is placed on the 3D printed lower jaw reconstruction model before surgery.

**Fig 8:**
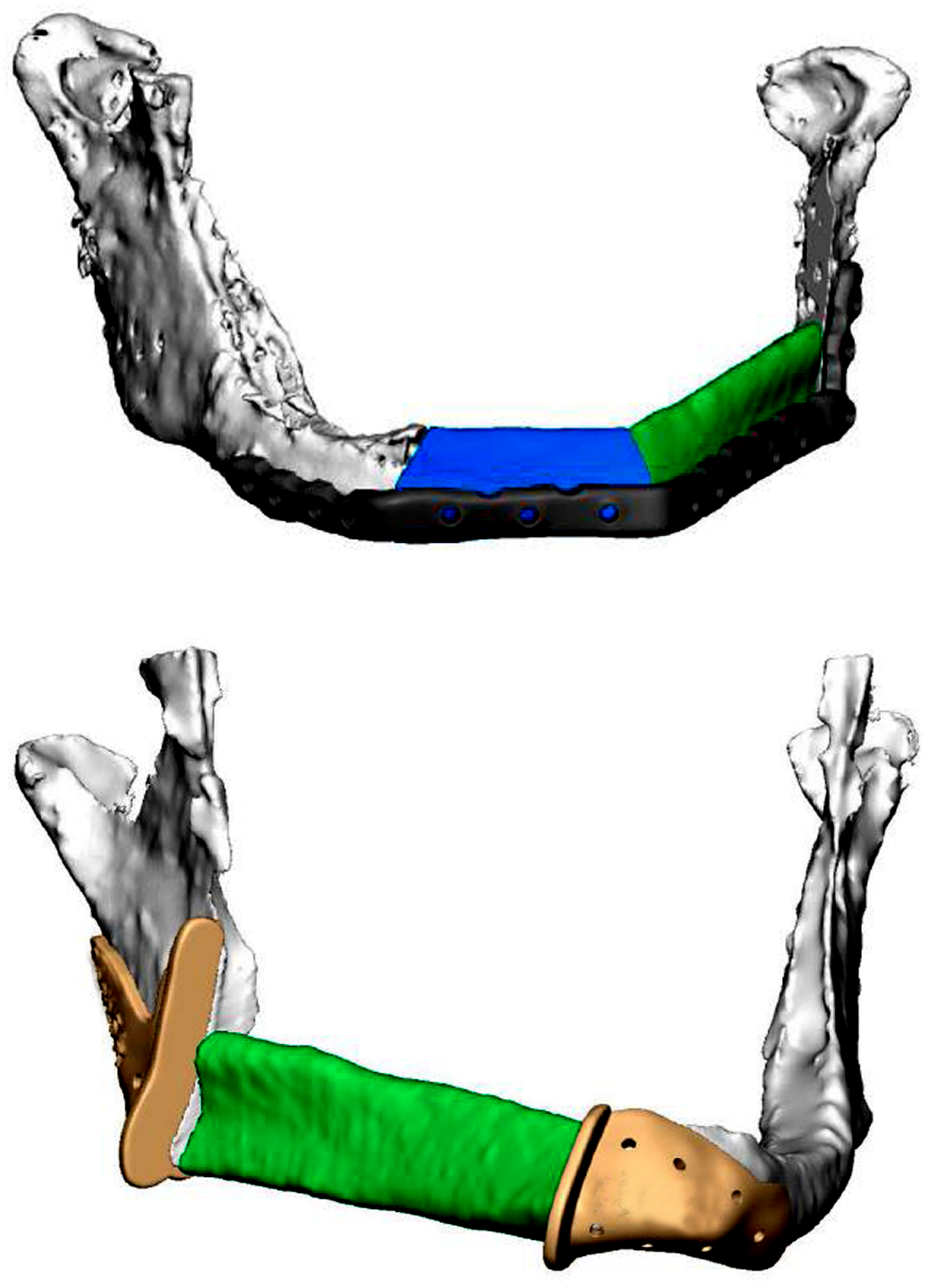
Virtual surgical plan of two cases. The 3D MRI-based lower jaw models (white), fibula segments (green and blue), patient specific reconstruction plate (grey, upper case) and the cutting guides (yellow, bottom case) of the first case (upper image) and second case (bottom image) are depicted.

Fig 7 shows the 3D printed PSP placed on the 3D printed reconstruction model. The virtual surgical reconstruction plans of both cases are shown in Fig 8.

**Fig 9:**
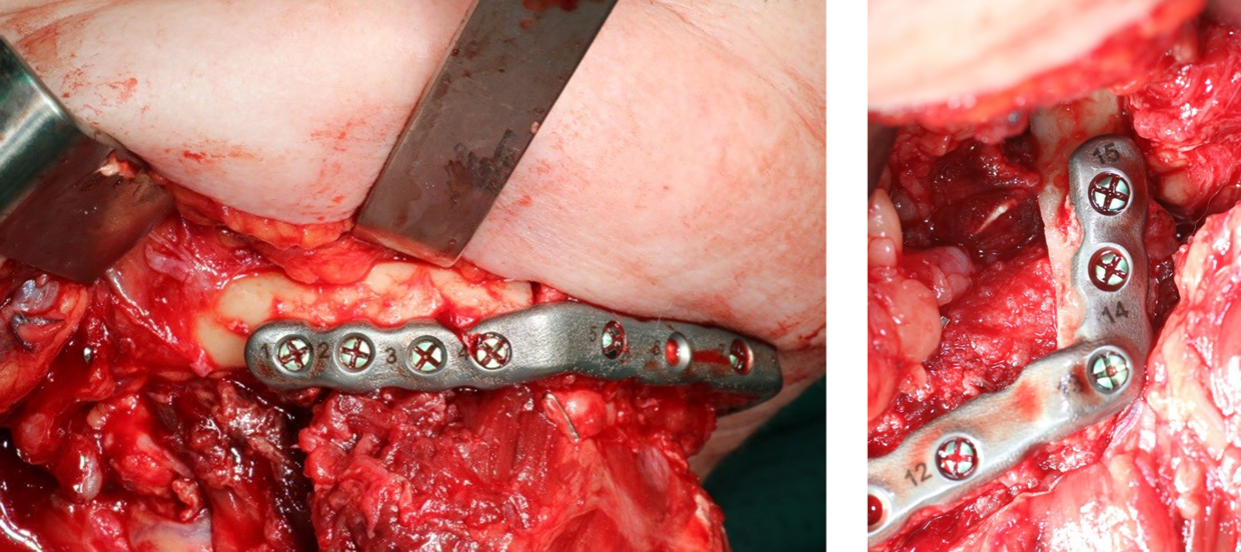
Patient specific reconstruction plate (PSP) in surgery. The PSP is connected to the fibula segments during surgery, with a perfect connection of fibula parts to the lower jaw.

Fig 9 shows the PSP connected to the two fibula segments during surgery. The fibula segments are connected to each other and to the original lower jaw bone without gaps and there is also no deviation visible between bone and plate.

The post-operative evaluation resulted in a mean distance of 2.3 mm between the midpoints of the saw planes. The mean distance between the centre points of the fibula segments was 3.8 mm and the mean angle between the axes of the fibula segments was 1.9° (S9 Fig).

## Discussion

This study is the first to report a 3D virtual planning workflow, based on an MRI Black Bone protocol for 3D lower jaw segmentation and resection planning.

Segmentation of the lower jaw is only possible when there is clear soft tissue-lower jaw contrast, the preference being minimal soft tissue contrast. Therefore, the flip angles were varied between 2° and 7° to compare the emanating tissue contrast. Application of quick FATSAT results in a nulled signal of fat (black in the images), leading to a more balanced contrast in the image and darker visualization of cancellous bone (less contrast between cortical and cancellous bone). Scanning was performed without interpolation to avoid rough 3D surfaces. GRAPPA is a parallel imaging technique, resulting in much shorter scanning times. This is especially beneficial in head and neck imaging, due to frequently occurring movement artefacts. This study showed that GRAPPA scans are faster in acquisition, however, not in segmentation time, since GRAPPA scans shows more noise. Finally, out of phase scanning was performed, since this results in small black lines on fat-water tissue borders. These black line artefacts are possibly useful for segmentation of the bones.

Based on the results of the test series, three sequences were selected to use in the validation series. A flip angle of 2° was chosen, in contrast to the 5° flip angle that *Eley et al*. used [25], with the knowledge that a smaller flip angle gives a more homogenous aspect with less soft tissue contrast.

The validation method (surface comparison with the gold standard 3D CT-based models) showed average deviation errors between 0.6 and 0.8 mm. Consequently, the coronoid processes of the lower jaw models were discarded, because these areas were difficult to separate from muscle attachments and they were also not relevant for the tumour resection planning and jaw reconstruction planning. Even though this reflects the limitation of segmenting 3D lower jaw models from MRI data, it was possible to plan surgical resection guides and reconstruction plates from the MRI data in patients.

The test series (phase 2) did not show any differences in segmentation quality between images obtained with GRAPPA, GRAPPA3 or without GRAPPA. However, the data of the validation series (phase 3) obtained with GRAPPA, required more extensive manual editing compared to the data scanned without the use of GRAPPA. More noise was observed in the GRAPPA images, one of the disadvantages of the faster scanning method and a possible explanation of bad segmentation quality. The impaired segmentation quality could also be explained by the fact that out of phase imaging and addition of FATSAT cancel out the effect of low soft tissue contrast caused by the low flip angle, as seen in Fig 4.

Marginal differences between the results of the validation series resulted in a hard selection process for the best sequence for segmentation and 3D modelling of the lower jaw. In most cases, the best segmentation was derived from the Black Bone sequence without the application of quick FATSAT and out of phase scanning. However, regarding the first clinical case, the sequence scanned with quick FATSAT and GRAPPA, proved to be suitable and accurate for this purpose as well. Since segmentation time is an important factor for application in clinical routines, the standard Black Bone sequence is preferred. It is advised to add the other sequences to the scanning protocol as well if time and situation permits, since the best sequence per case is not known at forehand.

To date, visualisation of tumour margins when planning a tumour resection of oral cancer requires fusion of CT data with MRI data [1]. CT data is used for 3D bone modelling, whereas the MRI is utilised to visualise the tumour. The resection guides are produced from the fused data. Even if multi-modality image fusion (CT and MRI) is not required, ‘fusion’ between MRI data is necessary, since the Black Bone sequence does not delineate the tumour. This ‘fusion’ of the MRI data from the same series is more accurate, since it is obtained from the same patient positioning and the same scanning moment.

Acquiring 3D lower jaw models from CT data is relatively simple and does not require long segmentation times. Creating 3D models based on MRI, however, take at least 3 times longer due to the required manual editing. The additional MRI-segmentation time is estimated to be 30 minutes per 3D lower jaw model for MRI-based segmentation. Since the scope of this research was a proof of principle in the use of MRI-based 3D models in surgical planning, the time gain is not proven in this study. However, we expect time gain, since MRI-MRI fusion might be faster than MRI-CT fusion, due to the same patient positioning. Moreover, when MRI based surgical planning is proven to be as accurate as CT-MRI based planning, the CT-scan is not necessary and this will save time and workload for the radiologist and the patient.

To our knowledge, this paper is the first to describe Black Bone MRI for 3D virtual resection margin planning. *Eley et al*. described the role of Black Bone MRI for radiation reduction in craniofacial imaging [25], for cephalometric analysis [26], and as a potential alternative to CT in 3D reconstruction of the craniofacial skeleton [27]. *Radetzki et al*. [28] used a Black Bone MRA sequence for virtual simulation and evaluation of femoralacetabular impingement. *Robinson et al*. [29] described the utility of Black Bone MRI in assessments of the foetal spine. The use of MRI for 3D virtual resection margin planning in head and neck oncologic reconstruction surgeries has not been reported before.

This study describes two successful cases where a patient specific reconstruction plate and lower jaw surgical guides were designed based on 3D MRI models. A prospective clinical trial must be performed to support the added value of the current 3D planning workflow in terms of improved resection margin planning, since evaluation of free bone margins is not included in this study. Besides evaluation of free bone margins, the prospective clinical trial can include cost comparison.

The focus of this study was on virtual lower jaw resection planning However, applying this method to the upper jaw is expected to be possible. Air and bone have identical grey values in Black Bone MRI, so segmentation difficulties are expected due to air in the sinuses connected to the upper jaw.

At this moment, we are not able to plan and resect soft tissue by guided surgery. All the above focuses on bony resection planning and bony margins. Complete tumour, bone marrow invasion, and tumour attached to the bone surface is visible on MRI data. This GTV is added to the bone model (now also MRI based) in order to plan the bony resection.

Using MRI data instead of CT data to produce 3D bone models of the lower jaw may eventually lead to more accurate margin planning, by avoiding the CT-MRI fusion step. The omission of data fusion will also lead to an optimised workflow. Since the planning workflow already consists of several steps, a shorter workflow based on one image modality is desirable and will contribute to optimised patient treatment.

The utilisation of the Black Bone sequence optimised and validated in this study, is not restricted to this specific workflow. The MRI sequence can be applied to various medical navigation planning software packages used for navigational surgery. Also, other medical fields (e.g. (maxillofacial) trauma surgery, orthopaedic surgery) might profit from these MRI sequences for bone visualisation.

## Conclusion

An MRI-based tumour delineation, bone segmentation and margin planning workflow was developed, using an optimised 3 Tesla Black Bone MRI head and neck protocol. This novel protocol allows tumour margin visualisation in pre-operative planning. The successful completion of two lower jaw, 3D MRI based planning, resection cases following reconstruction surgery is a first step in order to prove clinical feasibility.

## Supporting information

S1 Table Sequences and characteristics of the MRI sequences performed in the test series. FA = flip angle (degree), BW = pixel bandwidth (Hz/pixel), TR = Repetition time (ms), TE = Echo time (ms), TA = acquisition time (min).
(DOCX)

S2 Table 23 scientific articles utilised from the literature uncovered in the general exploratory phase. These articles all describe a method or methods for bone segmentation from MRI.
(DOCX)

S3 Table List of requirements for MRI sequence and settings and segmentation method. (DOCX)

S4 Table Evaluated sequences from the general exploratory phase including pixel size, slice thickness, and acquisition time. The comments column shows information about the segmentation process and quality.
(DOCX)

S1 Fig T1-weighted Dixon VIBE in phase sequence. Coronal, axial and sagittal slices and 3D model reconstruction of the lower jaw segmentation.
(TIF)

S2 Fig T1-weighted starVIBE sequence. (A) Coronal, axial and sagittal slices (left to right). (B) 3D model of the MRI-based (orange) with the CT-based lower jaw model (transparent). (C) Colour map showing the deviation between MRI- and CT-based model. The colour scale is from minus 6.0 mm (blue) to 6.0 mm (red).
(TIF)

S3 Fig T1-weighted TSE sequence. Coronal, axial and sagittal slices (left to right) and 3D model reconstruction of the lower jaw segmentation.
(TIF)

S4 Fig T2-weighted FLAIR + FATSAT sequence. Coronal, axial and sagittal slices (left to right) and 3D model reconstruction of the lower jaw segmentation.
(TIF)

S5 Fig T2-weighted blade sequence. Coronal, axial and sagittal slices (left to right) and 3D model reconstruction of the lower jaw segmentation.
(TIF)

S6 Fig Black bone VIBE sequence. (A) Coronal, axial and sagittal slices (left to right) showing the lower jaw. (B) 3D model of the segmented lower jaw. (C) Colour map showing the deviation between the MRI- and CT-based models of the segmented lower jaw.
(TIF)

S7 Fig MRI-based 3D lower jaw models utilised for surgery. Left: case 1, derived from black bone with quick FATSAT + GRAPPA and a flip angle of 2°. Right: case 2, derived from black bone with a flip angle of 2°.
(TIF)

S8 Fig Virtual CT-based lower jaw model with an MRI designed test plate. The plate shows small deviations from the CT-based model on the top side of the rami, the mental region, and the region where the masseter muscle overlaps the lower jaw.
(TIF)

S9 Fig Post-operative evaluation. Aligned planned (MRI-based, green) and post-operative (CBCT-based, blue) models. The fibula segment of the post-operative situation was replaced by the fibula segment of the plan. (A) Aligned models. (B) Cutting plane comparison. (C) Fibula segment axis and centre point comparison.
(JPG)

S1 File Validation series surface match alignment error. This file contains all alignment error values in mm per analysed patient.
(XLSX)

S2 File Validation series part to part comparison. This file contains all results of the part to part analysis in mm per analysed patient.
(XLSX)
