# Supplementary material for: Optimisation of three-dimensional lower jaw resection margin planning using a novel Black Bone magnetic resonance imaging protocol
Source: PLoS One. 2018 Apr 20;13(4):e0196059. doi: 10.1371/journal.pone.0196059 (PMC5909900; doi:10.1371/journal.pone.0196059)
Supplement: S1 Table — FA = flip angle (degree), BW = pixel bandwidth (Hz/pixel), TR = Repetition time (ms), TE = Echo time (ms), TA = acquisition time (min). (DOCX) [file pone.0196059.s001.docx]

**S1 Table. Sequences and characteristics of the MRI sequences performed in the test series.** FA = flip angle (degree), BW = pixel bandwidth (Hz/pixel), TR = Repetition time (ms), TE = Echo time (ms), TA = acquisition time (min)

| No. | Series description | FA | BW | TR | TE | TA | Volunteer |
| --- | --- | --- | --- | --- | --- | --- | --- |
| 1 | Standard | 2 | 210 | 6.2 | 2.53 | 2:34 | 1,2,3 |
| 2 | Standard | 3 | 210 | 6.2 | 2.53 | 2:34 | 1,2 |
| 3 | Out of phase | 5 | 500 | 3.78 | 1.54 | 1:34 | 1,2,3 |
| 4 | With quick FATSAT | 5 | 210 | 6.11 | 2.5 | 2:39 | 1,2,3 |
| 5 | Standard | 5 | 210 | 6.11 | 2.5 | 2:32 | 1,2,3 |
| 6 | Without interpolation | 5 | 210 | 6.2 | 2.53 | 4:13 | 1,2 |
| 7 | Standard | 7 | 210 | 6.2 | 2.53 | 2:34 | 1,2 |
| 8 | Without interpolation + GRAPPA | 5 | 210 | 6.2 | 2.52 | 3:28 | 2 |
| 9 | Out of phase + GRAPPA | 2 | 500 | 3.78 | 1.54 | 1:20 | 3 |
| 10 | Out of phase + GRAPPA3 | 2 | 500 | 3.78 | 1.54 | 58.37 | 3 |
| 11 | Out of phase | 2 | 500 | 3.78 | 1.54 | 2:26 | 3 |
| 12 | With quick FATSAT | 2 | 210 | 6.11 | 2.5 | 4:05 | 3 |
| 13 | With quick FATSAT + GRAPPA | 2 | 210 | 6.11 | 2.5 | 2:14 | 3 |
| 14 | With quick FATSAT + GRAPPA3 | 2 | 210 | 6.11 | 2.5 | 1:37 | 3 |
| 15 | With GRAPPA 3 | 2 | 210 | 6.11 | 2.5 | 1:34 | 3 |
| 16 | Out of phase + GRAPPA | 5 | 500 | 3.78 | 1.54 | 1:20 | 3 |
| 17 | With quick FATSAT +GRAPPA | 5 | 210 | 6.11 | 2.5 | 2:14 | 3 |
