# Supplementary material for: Optimisation of three-dimensional lower jaw resection margin planning using a novel Black Bone magnetic resonance imaging protocol
Source: PLoS One. 2018 Apr 20;13(4):e0196059. doi: 10.1371/journal.pone.0196059 (PMC5909900; doi:10.1371/journal.pone.0196059)
Supplement: S2 Table — These articles all describe a method or methods for bone segmentation from MRI. (DOCX) [file pone.0196059.s002.docx]

**S2 Table. 23 scientific articles utilised in the literature study of the general exploration phase.** These articles all describe a method or methods for bone segmentation from MRI.

| No. | Article | Author/Year |
| --- | --- | --- |
| 1 | Automatic graph-cut based segmentation of bones from knee magnetic resonance images for osteoarthritis research | *Ababneh et al.* 2011 |
| 2 | A 3D active model framework for segmentation of proximal femur in MR images | *Arezoomand et al.* 2015 |
| 3 | 3D representation of the surface topography of normal and dysplastic trochlea using MRI. | *Biedert et al.* 2011 |
| 4 | MR image segmentation of the knee bone using phase information | *Bourgeat et al.* 2007 |
| 5 | Focused shape models for hip joint segmentation in 3D magnetic resonance imaging | *Chandra et al*. 2014 |
| 6 | Measuring bone erosion and edema in rheumatoid arthritis: a comparison of manual segmentation and RAMRIS methods | *Crowley et al.* 2011 |
| 7 | A fully automated human knee 3D MRI bone segmentation using the ray casting technique | *Dodin et al.* 2011 |
| 8 | Segmentation of skull and scalp in 3-D human MRI using mathematical morphology | *Dogdas et al*. 2005 |
| 9 | 3D MRI Analysis of the Lower Legs of Treated Idiopathic Congenital Talipes Equinovarus (Clubfoot) | *Duce et al.* 2013 |
| 10 | “Black Bone” MRI: a potential alternative to CT with three-dimensional reconstruction of the craniofacial skeleton in the diagnosis of craniosynostosis | *Eley et al. 2014* |
| 11 | Segmentation of the bones in MRIs of the knee using phase, magnitude and shape information | *Fripp et al.* 2007 |
| 12 | The accuracy of 3-dimensional magnetic resonance 3D vibe images of the mandible: an in vitro comparison of magnetic resonance imaging and computed tomography | *Goto et al.* 2007 |
| 13 | 3DMR osseous reconstructions of the shoulder using a gradient-echo based two-point Dixon reconstruction: a feasibility study | *Gyftopoulos et al*. 2013 |
| 14 | MIDA: A Multimodal Imaging-Based Detailed Anatomic Model of the human head and neck | *Iacono et al.* 2015 |
| 15 | A two-stage rule-constrained seedless region growing approach for mandibular body segmentation in MRI | *Ji et al*. 2013 |
| 16 | A fully automated trabecular bone structural analysis tool based on T2*-weighted magnetic resonance imaging | *Kraiger et al.* 2012 |
| 17 | T1/T2*-weighted MRI provides clinically relevant pseudo-CT density data for the pelvic bones in MRI-only based radiotherapy treatment planning | *M. Kapanen & M. Tenhunen* 2013 |
| 18 | Quantification of the accuracy of MRI generated 3D models of long bones compared to CT generated 3D models | *Rathnayaka et al.* 2012 |
| 19 | Segmentation of the skull in MRI volumes using deformable model and taking the partial volume effect into account. | *Rifa et al.* 2000 |
| 20 | Extreme leg motion analysis of professional ballet dancers via MRI segmentation of multiple leg postures | *Schmid et al.* 2011 |
| 21 | Robust statistical shape models for MRI bone segmentation in presence of small field of view | *Schmid et al.* 2011 |
| 22 | Unsupervised segmentation and quantification of anatomical knee features: Data from the osteoarthritis initiative | *Tames-Pena et al*. 2012 |
| 23 | Segmentation accuracy of long bones | *Van den Broeck et al.* 2014 |
| 24 | Segmentation of bones in magnetic resonance images of the wrist | *Wlodarczyk et al.* 2015 |
