# Supplementary material for: Optimisation of three-dimensional lower jaw resection margin planning using a novel Black Bone magnetic resonance imaging protocol
Source: PLoS One. 2018 Apr 20;13(4):e0196059. doi: 10.1371/journal.pone.0196059 (PMC5909900; doi:10.1371/journal.pone.0196059)
Supplement: S3 Table — (DOCX) [file pone.0196059.s003.docx]

**S3 Table. List of requirements for MRI sequence and settings and segmentation method**

| Requirements for MRI sequence and settings: |
| --- |
| 1. Contrast between bone and surrounding tissue |
| 1. Executable in 3 Tesla MRI scanner |
| 1. Isotropic voxel size ≤ 1mm |
| 1. 3D acquisition |
| 1. Mandible in field of view |
| 1. Acquisition time ≤ 10 minutes |

| Requirements for the segmentation method: |
| --- |
| 1. Segmentation time ≤ 1 hour |
| 1. Software available in the hospital |
| 1. Maximal deviation from ‘gold standard’ at critical sites ≤ 1mm |
| 1. No fusion of different imaging modalities |
| 1. Result is an 3D STL file |
| 1. Compatible with MRI data |
| 1. Applicable in all individual cases |
