# Supplementary material for: Optimisation of three-dimensional lower jaw resection margin planning using a novel Black Bone magnetic resonance imaging protocol
Source: PLoS One. 2018 Apr 20;13(4):e0196059. doi: 10.1371/journal.pone.0196059 (PMC5909900; doi:10.1371/journal.pone.0196059)
Supplement: S4 Table — The comments column shows information about the segmentation process and quality. (DOCX) [file pone.0196059.s004.docx]

**S4 Table. Evaluated sequences of general exploration phase including pixel size, slice thickness, and acquisition time.** The comments column shows information about the segmentation process and quality.

| Sequence | Pixel size (mm) | Slice thickness (mm) | Acquisition time (min) | Comments | Figure |
| --- | --- | --- | --- | --- | --- |
| T1 3D VIBE | 1.0 x 1.0 | 1.0 | 06:34 | Segmentation n/a, cadaver scan |  |
| T1-weighted 3D Dixon VIBE in phase | 0.7 x 0.7 | 0.7 | 06:23 | Promising segmentation | S5 Figure |
| T1-weighted 3D StarVIBE | 0.9 x 0.9 | 2.0 | 02:53 | Segmentation difficulties due to 2.0 mm slice thickness, a lot of manual editing | S6 Figure |
| T1-weighted 3D MPRAGE | 1.0 x 1.0 | 1.0 | 05:24 | Segmentation n/a, cadaver scan |  |
| T1-weighted TSE | 0.7 x 0.7 | 3.0 | 01:24*2 | Bad segmentation quality, due to 3.0 slice thickness | S7 Figure |
| T2-weighted 3D FLAIR + FATSAT | 1.0 x 1.0 | 1.0 | 05:55 | Tolerable segmentation quality, noisy | S8 Figure |
| T2-weighted Blade | 0.6 x 0.6 | 3.0 | 01:31*2 | Bad segmentation quality due to bad resolution in coronal and sagittal slices | S9 Figure |
| 3D black bone VIBE | 0.5 x 0.6 | 1.0 | 06:50 | Promising segmentation | S10 Figure |
