# Supplementary figures and images for: Optimisation of three-dimensional lower jaw resection margin planning using a novel Black Bone magnetic resonance imaging protocol
Source: PLoS One. 2018 Apr 20;13(4):e0196059. doi: 10.1371/journal.pone.0196059 (PMC5909900; doi:10.1371/journal.pone.0196059)

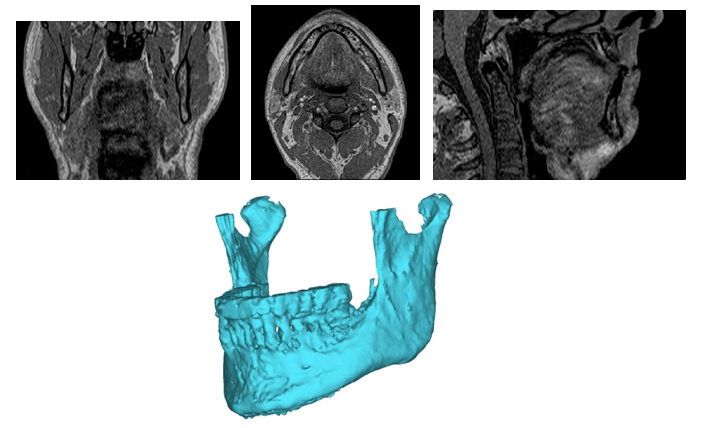

Supplement: S1 Fig — Coronal, axial and sagittal slices and 3D model reconstruction of the lower jaw segmentation. (TIF) [file pone.0196059.s005.tif]

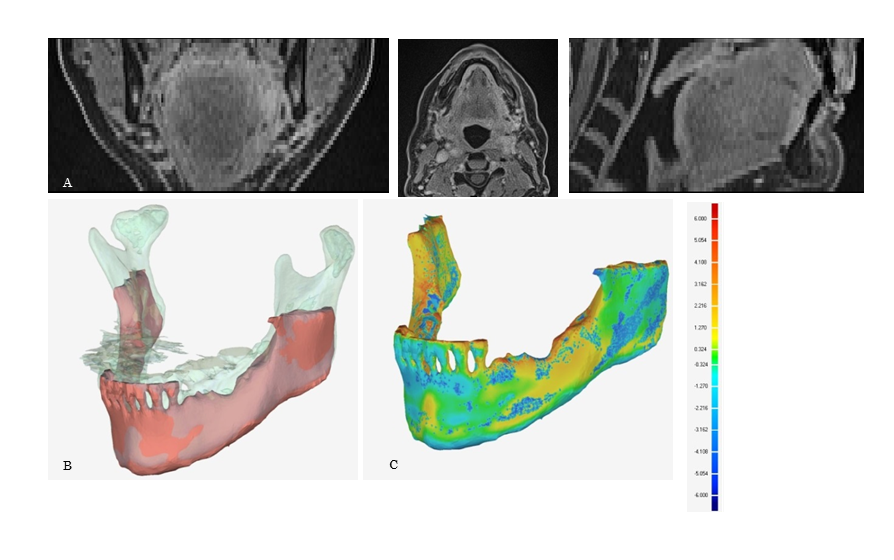

Supplement: S2 Fig — (A) Coronal, axial and sagittal slices (left to right). (B) 3D model of the MRI-based (orange) with the CT-based lower jaw model (transparent). (C) Colour map showing the deviation between MRI- and CT-based model. The colour scale is from minus 6.0 mm (blue) to 6.0 mm (red). (TIF) [file pone.0196059.s006.tif]

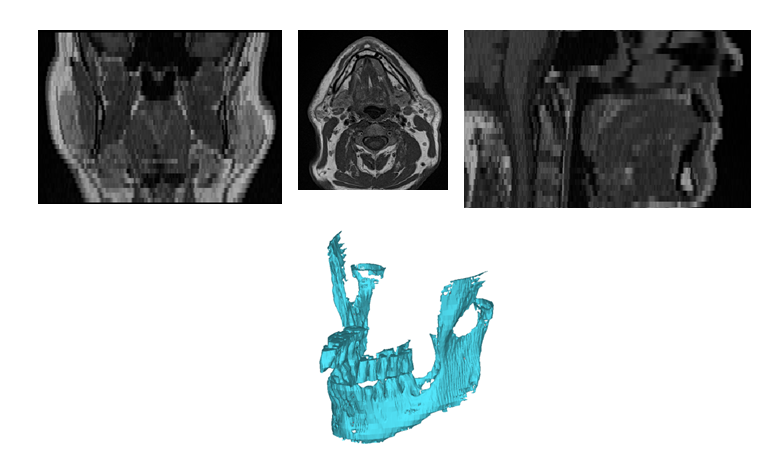

Supplement: S3 Fig — Coronal, axial and sagittal slices (left to right) and 3D model reconstruction of the lower jaw segmentation. (TIF) [file pone.0196059.s007.tif]

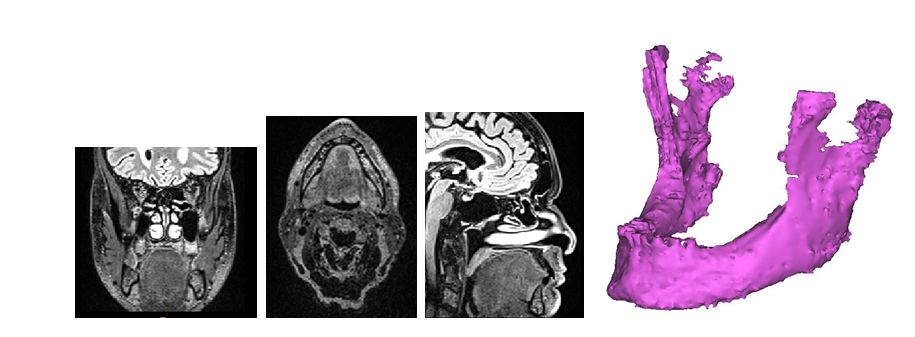

Supplement: S4 Fig — Coronal, axial and sagittal slices (left to right) and 3D model reconstruction of the lower jaw segmentation. (TIF) [file pone.0196059.s008.tif]

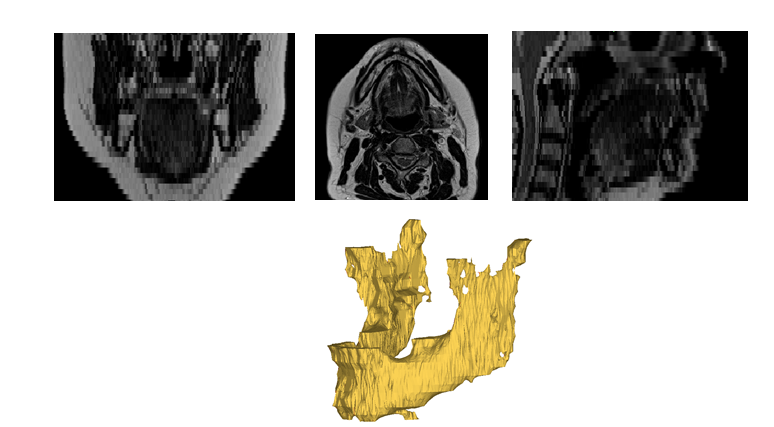

Supplement: S5 Fig — Coronal, axial and sagittal slices (left to right) and 3D model reconstruction of the lower jaw segmentation. (TIF) [file pone.0196059.s009.tif]

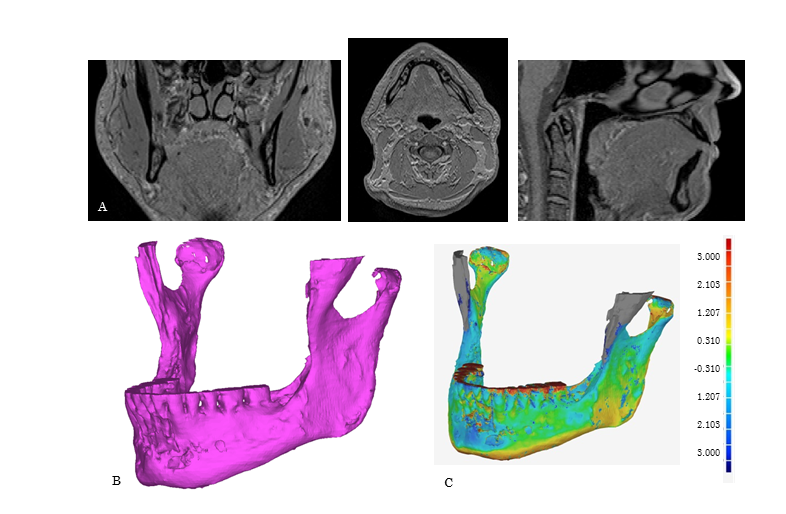

Supplement: S6 Fig — (A) Coronal, axial and sagittal slices (left to right) showing the lower jaw. (B) 3D model of the segmented lower jaw. (C) Colour map showing the deviation between the MRI- and CT-based models of the segmented lower jaw. (TIF) [file pone.0196059.s010.tif]

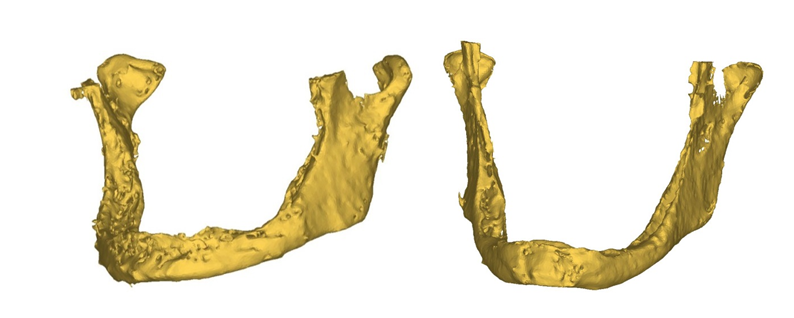

Supplement: S7 Fig — Left: case 1, derived from black bone with quick FATSAT + GRAPPA and a flip angle of 2°. Right: case 2, derived from black bone with a flip angle of 2°. (TIF) [file pone.0196059.s011.tif]

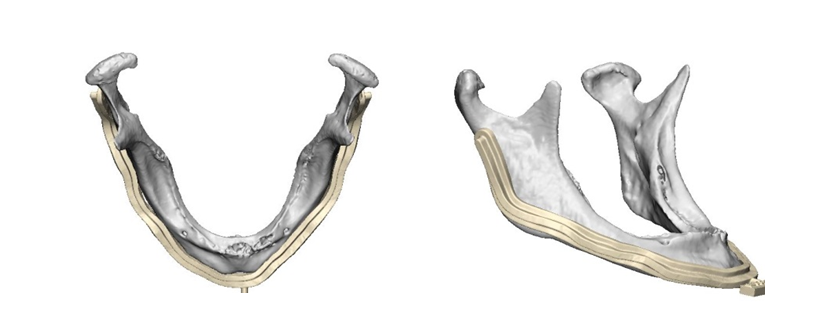

Supplement: S8 Fig — The plate shows small deviations from the CT-based model on the top side of the rami, the mental region, and the region where the masseter muscle overlaps the lower jaw. (TIF) [file pone.0196059.s012.tif]

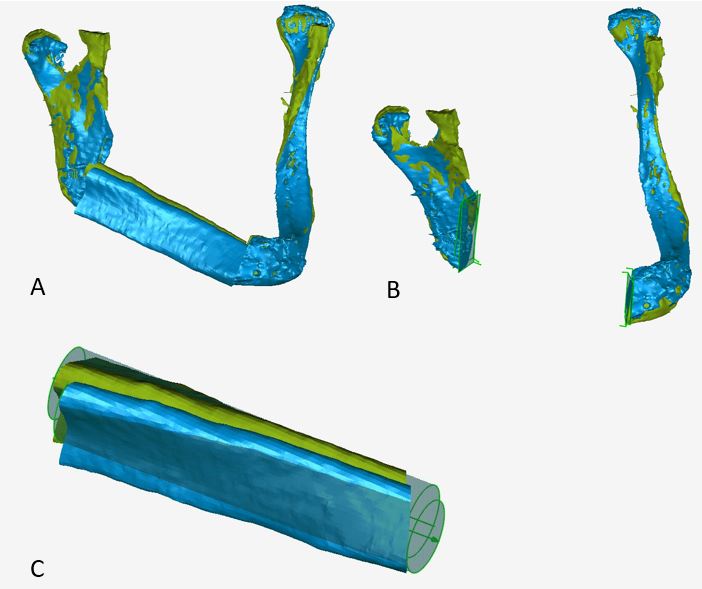

Supplement: S9 Fig — Aligned planned (MRI-based, green) and post-operative (CBCT-based, blue) models. The fibula segment of the post-operative situation was replaced by the fibula segment of the plan. (A) Aligned models. (B) Cutting plane comparison. (C) Fibula segment axis and centre point comparison. (JPG) [file pone.0196059.s013.JPG]
